# Potential Causal Relationships Between Circulating Micronutrient Levels and Multiple Neuroimmune Diseases: A Genetic Association Analysis

**DOI:** 10.1002/brb3.70848

**Published:** 2025-09-09

**Authors:** Longhao Chen, Xuzhou Wu, Kaizheng Wang, Xingchen Zhou, Yika Mou, Zhen Liu, Zhifang Shen, Zhizhen Lv, Lijiang Lv

**Affiliations:** ^1^ The Third Affiliated Hospital of Zhejiang Chinese Medical University Hangzhou China; ^2^ The Third School of Clinical Medicine (School of Rehabilitation Medicine) Zhejiang Chinese Medical University Hangzhou China; ^3^ Research Institute of Tuina (Spinal Disease) Zhejiang Chinese Medical University Hangzhou China; ^4^ First Affiliated Hospital of Guangxi University of Chinese Medicine Nanning China; ^5^ Jiaxing Hospital of Traditional Chinese Medicine Jiaxing China

**Keywords:** causality, genome‐wide association study, micronutrients, neuroimmune diseases

## Abstract

**Background:**

Growing evidence suggests a close association between circulating micronutrient levels and neuroimmune diseases. Nevertheless, the causal relationship between them remains unclear. Furthermore, due to confounding factors, many micronutrients implicated in these diseases remain unidentified. This study aimed to determine the causal relationship between circulating micronutrients and neuroimmune diseases through genetic association analysis, and to analyze the regulatory role of circulating micronutrients in neuroimmune diseases.

**Method:**

In this study, we used a two‐sample mendelian randomization (MR) analysis to explore the causal relationship between micronutrients levels and neuroimmune disease. Fourteen micronutrients were screened from a published genome‐wide association study (GWAS). Neuroimmune diseases include multiple sclerosis (MS), Guillain–Barre syndrome (GBS), acute disseminated encephalomyelitis (ADEM), acute poliomyelitis (AP), sequelae of poliomyelitis (SP), optic neuritis (ON), and myasthenia gravis (MG). Data on these seven neuroimmune diseases came from the FinnGen database and included 5523 cases and 2,860,006 controls. The inverse variance weighting (IVW) method was used as the main MR analysis method, and sensitivity analysis was performed to determine MR hypotheses.

**Results:**

Through MR analysis and sensitivity testing, we identified significant causal relationships between four neuroimmune diseases and micronutrient levels. Specifically, MS was causally associated with magnesium levels (OR: 0.467, 95% CI: 0.269–0.809, *p* = 0.007), ADEM with folate levels (OR: 0.022, 95% CI: 0.001–0.957, *p* = 0.047), ON with vitamin B6 levels (OR: 0.382, 95% CI: 0.187–0.778, *p* = 0.008), and MG with iron levels (OR: 0.194, 95% CI: 0.043–0.867, *p* = 0.032). Sensitivity analysis showed that there was no level pleiotropic or heterogeneity in our study results.

**Conclusion:**

This study established the causal relationship between micronutrients and neuroimmune diseases. These findings provide new insights into the etiology of neuroimmune diseases and provide a theoretical basis for micronutrient regulation, prevention, and treatment of neuroimmune diseases.

## Graphical abstract

Mendelian randomization analysis identified causal relationships between specific micronutrients (magnesium, folate, vitamin B6, iron) and neuroimmune diseases (MS, ADEM, ON, MG). These findings provide new insights into disease mechanisms and support micronutrient modulation as a potential preventive or therapeutic strategy.

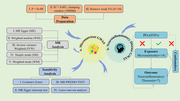

## 1 Introduction

Over the past three decades, the global incidence and prevalence of immune‐mediated neurological disorders have steadily increased (Sterling et al. 2022) (Cohen et al. 2024). These disorders, characterized by aberrant immune responses targeting components of the nervous system, include multiple sclerosis (MS), Guillain–Barré syndrome (GBS), acute disseminated encephalomyelitis (ADEM), acute poliomyelitis (AP), sequelae of poliomyelitis (SP), optic neuritis (ON), and myasthenia gravis (MG). Among these, ADEM primarily affects children, while MS, GBS, AP, SP, and ON predominantly occur in younger individuals. In contrast, MG is more common in the elderly population (Oh et al. 2018; Dobson and Giovannoni 2019; Langille 2023; Wolska‐Krawczyk 2022; Kidd et al. 1996; Toosy et al. 2014; Breiner et al. 2016). Neuroimmune diseases trigger a complex and multi‐faceted harmful process on the white and gray matter of the brain, thus affecting people's cognitive levels and mental state (Schmitz et al. 2023). Globally, neuroimmune diseases have affected the normal lives of tens of millions of people. To date, most agents have failed to demonstrate efficacy in clinical trials targeting cellular damage associated with neuroimmune diseases (Pizcueta et al. 2023). The global health field is urgently seeking corresponding effective prevention and treatment measures due to the surge in treatment costs and harm of neuroimmune diseases.

Micronutrients, comprising vitamins and trace elements, are a category of low‐dose nutrients essential for maintaining normal physiological functions in organisms (Berger et al. 2022). They are widely distributed in natural environments such as the earth's crust and seawater, as well as diverse food sources. These substances play indispensable roles in biological processes including growth and development, antioxidant defense, immune regulation, and modulation of inflammatory responses (Maggini et al. 2018). Recent evidence indicates that imbalances in micronutrients are closely associated with the pathogenesis of various diseases. The underlying mechanisms potentially involve disordered cellular metabolism, oxidative stress imbalance, and immune dysfunction (Michael et al. 2022) (Barra et al. 2021). Many countries attach great importance to the impact of micronutrients on diseases and have corresponding measurements and statistics on disease micronutrients in patient groups, in order to guide the construction of a healthy environment and the understanding, diagnosis, and treatment of diseases through scientific statistical results (Kudabayeva et al. 2017) (Martusevich and Karuzin 2019) (Taghizadeh et al. 2023).

Recent research has proposed a potential relationship between micronutrients and the development of neuroimmune diseases. Micronutrients are involved in the occurrence and development of immune diseases of the nervous system and can even regulate treatment and prognosis. Wu et al. (2022) reported that the trace element Se can relieve neuroinflammation in mice. The prevalence of MS is related to boron and manganese in the environment and trace elements in the diet (Zielińska and Michońska 2022) (Zapadniuk 1992). The inflammatory process of ON may be related to the disorder of the homeostasis of Cd, Cu, and Fe (Kaźmierczak et al. 2014). Vitamin families such as vitamin D have been shown to have regulatory and therapeutic effects with MS, GBS, MG (Tarasiuk et al. 2019) (Sharma et al. 2022) (Larrosa‐Domínguez 2023), vitamin B12 and AP, SP, and ON (DAUR, 1954). Micronutrients can directly participate in the regulation of cellular immunity and tissue healing in the central nervous system or use substances produced through their induction to reshape and improve (Lahoda Brodska et al. 2023).

Based on the aforementioned research data and observations, Mendelian randomization (MR) offers a promising approach for exploring causal relationships. MR was originally proposed by Smith and Ebrahim (2003) to use genetic variation as a tool variable to eliminate confounding bias and reverse causality (Greco et al. 2015). In this study, we collected data on micronutrients and neuroimmune diseases from the genome‐wide association study (GWAS) database and used MR analysis to reveal the causal relationship between them. This study aims to provide innovative insights into the prevention and treatment of neuroimmune diseases by elucidating the complex interactions between micronutrients and neuroimmune disorders.

## 2 Materials and Methods

### 2.1 Study Design

**FIGURE 1:**
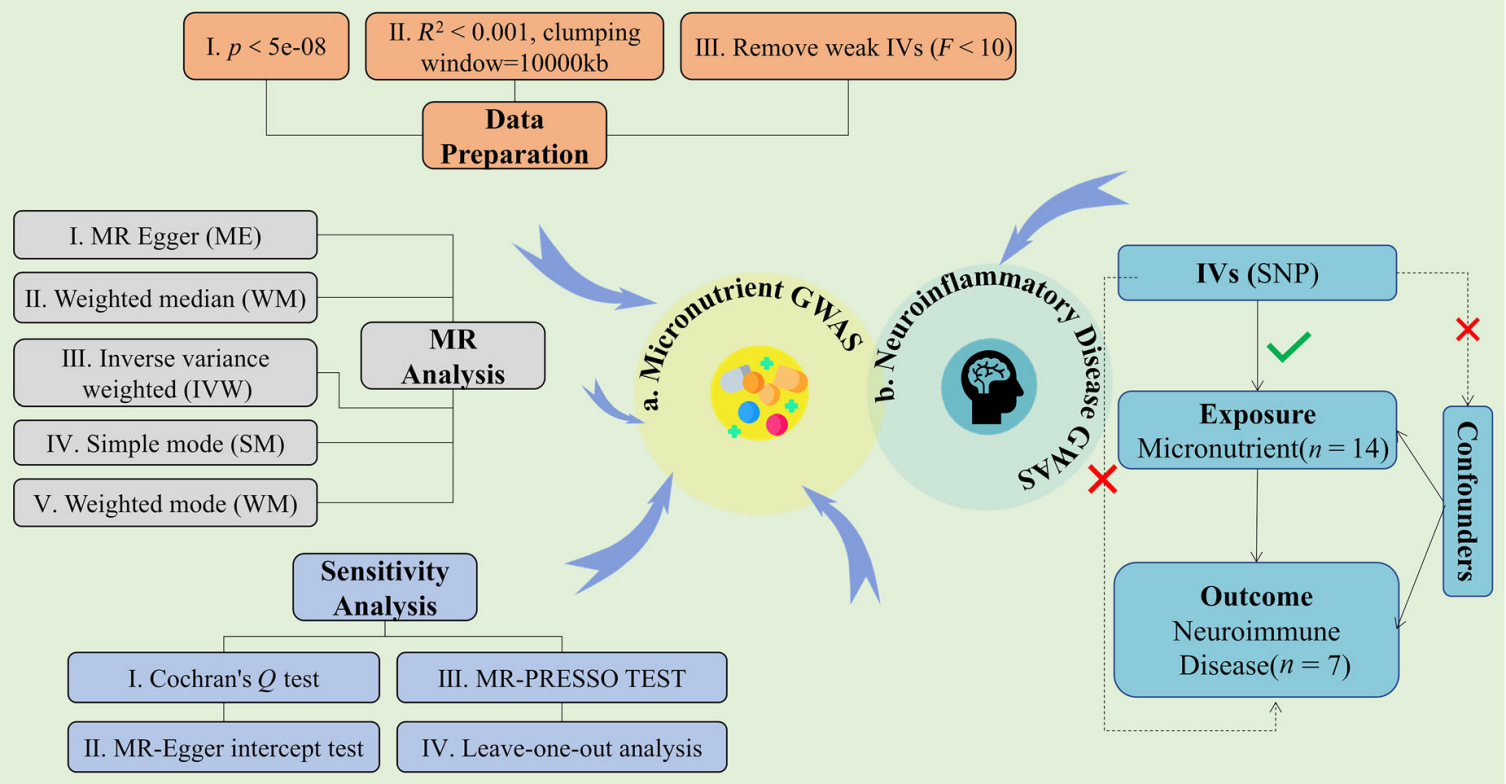
Overview of present MR analyses and assumptions. This figure systematically illustrates the complete Mendelian randomization (MR) research workflow from instrumental variable (IVs) selection to causal effect analysis, encompassing key steps including data preprocessing, multi‐method MR analysis, and sensitivity testing. LD: linkage disequilibrium.

This study was designed based on the STROBE‐MR report (Skrivankova et al. 2021). Figure 1 shows a schematic diagram of the study design. We conducted a two‐sample MR study using publicly summarized statistical data from GWAS. Both exposure and outcome cohorts were limited to subjects of European descent to reduce bias in population stratification (Sanderson et al. 2022). The MR analysis was based on three key assumptions: (1) instrumental variables (IVs) are closely related to circulating micronutrient concentrations, (2) IVs should not be influenced by known or unknown confounding factors, and (3) IVs affect neuroimmune disease only through circulating micronutrient concentrations. All data used in this study came from studies that received the consent and ethical approval of the relevant participants, so ethical approval from the Institutional Review Board was not required for this study.

### 2.2 Circulating Micronutrient Genetic Data

We searched the GWAS catalog and PubMed for published GWAS evaluating individuals of European descent. The last search was conducted in 2024. In total, we identified 14 micronutrients of potential interest: copper, calcium, carotene, folate, iron, magnesium, potassium, selenium, vitamin B12, vitamin B6, vitamin C, vitamin D, vitamin E, and zinc.

### 2.3 Neuroimmune Diseases Genetic Data

We used neuroimmune diseases as outcome data. This study included seven neuroimmune diseases: MS, GBS, ADEM, AP, SP, ON, and MG. The data of the above seven neuroimmune diseases are all from the FinnGen database, and the samples included are all public summary statistics of European ancestry cohorts. In the FinnGen database, cases and controls are defined according to the international classification of disease codes in hospital records. Meta‐analysis was performed for the summary statistics of each neuroimmune disease. The outcome data information is shown in Table 1.

**TABLE 1:** Source of outcome genome‐wide association study summary data.

No.	GWAS ID	Year	Trait	Cases	Controls	Number of SNPs	Population
1	finn‐b‐G6_MS	2021	Multiple sclerosis	2409	408,561	16,380,460	European
2	finn‐b‐G6_GUILBAR	2021	Guillain–Barre syndrome	445	405,136	16,380,463	European
3	finn‐b‐G6_DISSOTH	2021	Acute disseminated encephalomyelitis	300	409,304	16,380,465	European
4	finn‐b‐AB1_POLIOMYELITIS	2021	Acute poliomyelitis	396	409,849	16,380,460	European
5	finn‐b‐AB1_SEQULAE_POLIO	2021	Sequelae of poliomyelitis	217	409,536	16,380,466	European
6	finn‐b‐H7_OPTNEURITIS	2021	Optic neuritis	1295	409,190	16,380,463	European
7	finn‐b‐G6_MYASTHENIA	2021	Myasthenia gravis	461	408,430	16,380,458	European

*Note*: This table summarizes the genome‐wide association studies (GWAS) used in the analysis.

Abbreviations: GWAS: genome‐wide association study; SNPs: single‐nucleotide polymorphisms.

### 2.4 Instrumental Variables

(1) SNP screening criteria: SNPs with a *p* value less than 5e‐08 were selected. (2) Sample data source: we used the 1000 European genomes project samples as reference data. (3) LD screening: within the 10,000 kb range of the genome, we screened out those SNPs with an LD value (*R* ^2^) less than 0.001, ensuring that the linkage relationship between them is weak. (4) Intra‐block SNP selection: for each LD block, we retained the SNP with the smallest *p* value to improve the significance of the results. (5) F‐statistic screening: we calculated the F‐statistic for each SNP and eliminated SNPs with *F*‐values ​​less than 10 to avoid the problem of insufficient statistical power (Cai et al. 2022) (K. W. Choi et al. 2019). Finally, the selected IVs are listed in Supporting Information S1.

### 2.5 The Method and Explanation of MR Analysis

In MR analysis, a variety of methods are used to assess causal relationships. The main method used is the inverse variance weighting (IVW), combined with weighted median, MR Egger, simple mode, and weighted mode, which are auxiliary analysis tools. The IVW method is used as the preferred method, while other methods are supplemented and verified according to different assumptions and data characteristics (Bowden et al. 2015). These methods have different assumptions when applied. The IVW method assumes that all IVs have no horizontal pleiotropy, while the weighted median rule is applicable to cases where there is a small amount (no more than 50%) of horizontal pleiotropy. The weighted median method is considered to be a more reliable choice for detected heterogeneity without involving pleiotropy (Bowden et al. 2016). MR‐Egger regression is used to test horizontal pleiotropy, especially when most IVs may be affected by pleiotropy. The simple model estimates the causal effect through a single genetic variant, which is suitable for dealing with cases where heterogeneity appears in the data, but there is no systematic bias. The weighted model introduces weights on the basis of the simple model, so that each effect estimate is weighted according to its importance, so it is more effective when most IVs perform consistently.

In the process of causal inference, IVW is used as the primary analysis tool, especially for the assumption that there is no horizontal pleiotropy. In addition, MR‐Egger regression and MR‐PRESSO test are commonly used methods to identify outliers in horizontal pleiotropy. If the *p* value of MR‐Egger regression exceeds 0.05, it can be considered that there is no significant horizontal pleiotropy. In order to assess whether there is heterogeneity in IVs, the Cochran's *Q* test is used. If the *p* value is less than 0.05, it means that there is heterogeneity, and the IVW method should be used to obtain a more conservative estimate. We also conducted a leave‐one‐out sensitivity analysis to detect possible outliers and the stability of the results. The *p* value in the analysis has been corrected for multiple tests. MR statistical results are shown in Supporting Information S2. The results of sensitivity tests are shown in Supporting Information S3/4.

All analyses were performed in the R programming environment, and the main tools used included the “VariantAnnotation” package, the “mrcieu/gwasglue” package, and the “MRCIEU/TwoSampleMR” package.

## 3 Results

### 3.1 MR Analysis Results of MS and 14 Micronutrients

**FIGURE 2:**
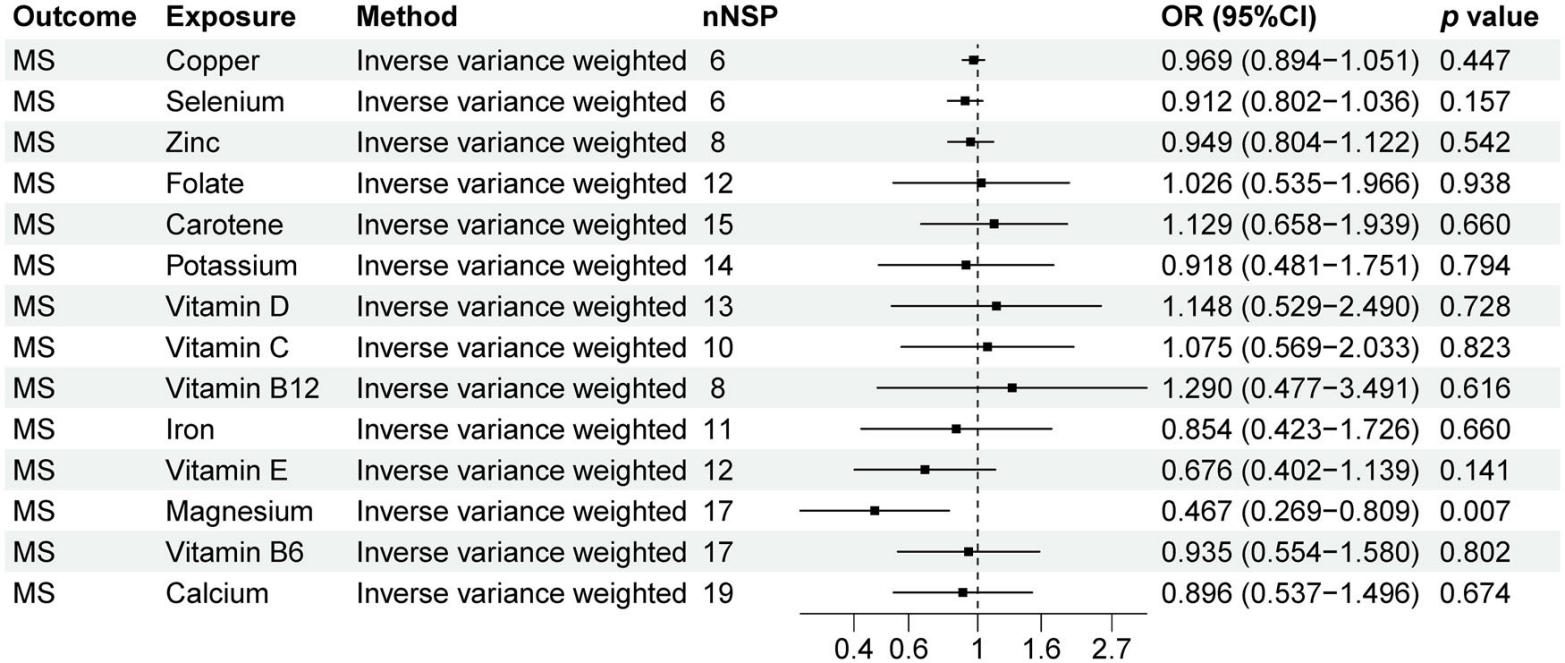
MR analysis results of MS and 14 micronutrients. Forest plot displays the causal associations between multiple sclerosis (MS) and 14 micronutrients using inverse‐variance weighted (IVW) method. CI: confidence interval; nSNP: number of single‐nucleotide polymorphisms used as instrumental variables; OR: odds ratio.

As shown in Figure 2, we can observe a causal relationship between MS and 14 micronutrients levels. We found a causal relationship between MS and Magnesium levels (odds ration [OR]: 0.467, 95% confidence interval [CI]: 0.269–0.809, *p* = 0.007). The scatter plot in Figure 9A clearly shows the linear relationship between SNP effect values and MS and magnesium levels, suggesting that magnesium levels may be a protective factor for MS. Our study showed that there is no causal relationship between MS and copper, calcium, carotene, folate, iron, potassium, selenium, vitamin B12, vitamin B6, vitamin C, vitamin D, vitamin E, and zinc (*p* > 0.05).

### 3.2 MR Analysis Results of GBS and 14 Micronutrients

**FIGURE 3:**
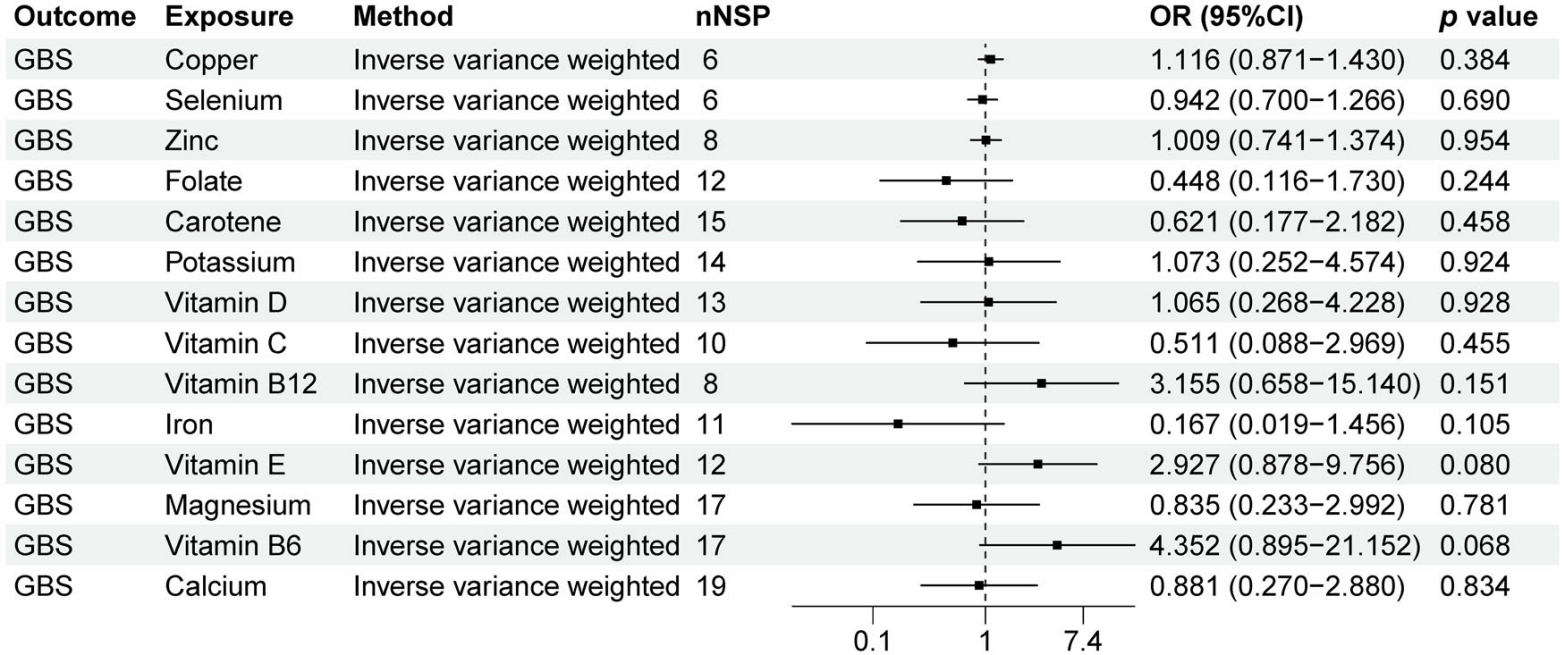
MR analysis results of GBS and 14 micronutrients. Forest plot displays the causal associations between Guillain–Barré syndrome (GBS) and 14 micronutrients using inverse‐variance weighted (IVW) method. CI: confidence interval; nSNP: number of single‐nucleotide polymorphisms used as instrumental variables; OR: odds ratio.

As shown in Figure 3, we can observe a causal relationship between GBS and 14 micronutrients levels. Our study showed that there is no causal relationship between GBS and copper, calcium, carotene, folate, iron, magnesium, potassium, selenium, vitamin B12, vitamin B6, vitamin C, vitamin D, vitamin E, and zinc (*p* > 0.05).

### 3.3 MR Analysis Results of ADEM and 14 Micronutrients

**FIGURE 4:**
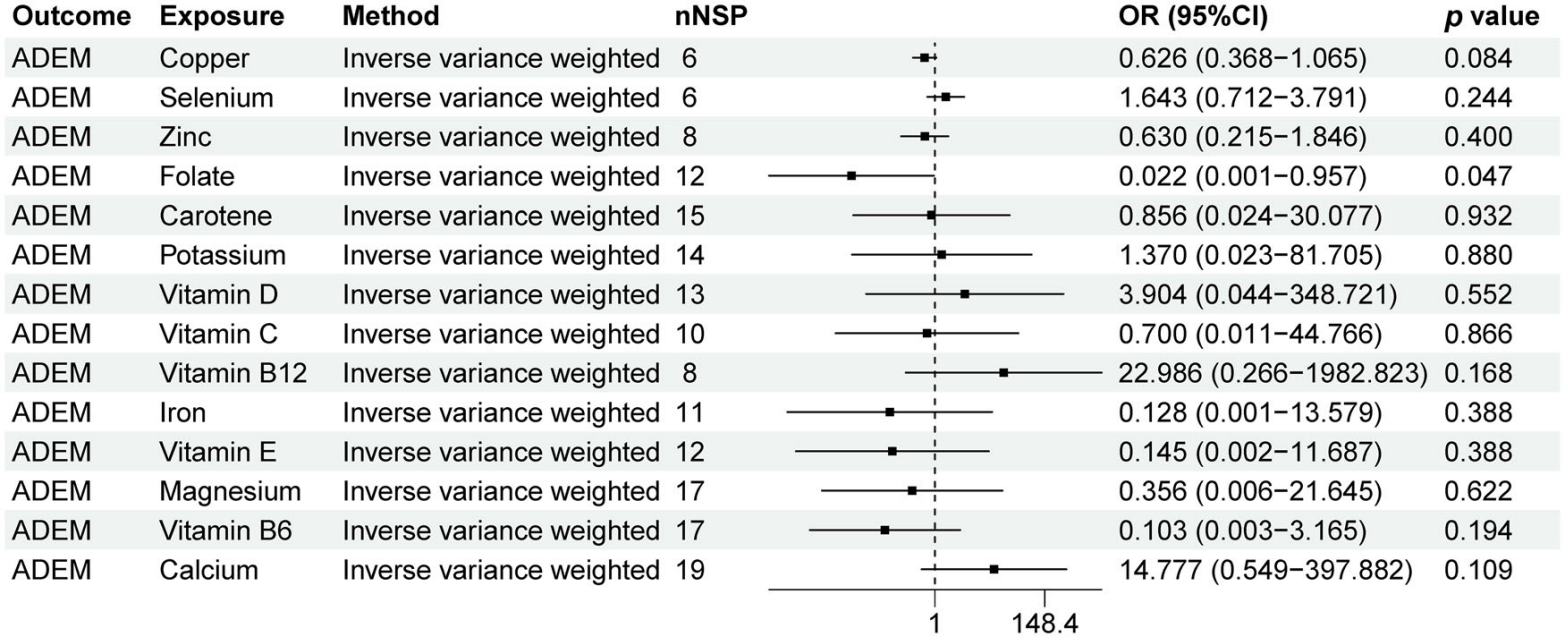
MR analysis results of ADEM and 14 micronutrients. Forest plot displays the causal associations between acute disseminated encephalomyelitis (ADEM) and 14 micronutrients using inverse‐variance weighted (IVW) method. CI: confidence interval; nSNP: number of single‐nucleotide polymorphisms used as instrumental variables; OR: odds ratio.

As shown in Figure 4, we can observe a causal relationship between ADEM and 14 micronutrients levels. There is a causal relationship between ADEM and folate levels (OR: 0.022, 95% CI: 0.001–0.957, *p* = 0.047). The scatter plot in Figure 9B clearly shows the linear relationship between SNP effect values and ADEM and folate levels, suggesting that folate levels may be a protective factor for ADEM. Our study showed that there is no causal relationship between ADEM and copper, calcium, carotene, iron, magnesium, potassium, selenium, vitamin B12, vitamin B6, vitamin C, vitamin D, vitamin E, and zinc (*p* > 0.05).

### 3.4 MR Analysis Results of AP and 14 Micronutrients

**FIGURE 5:**
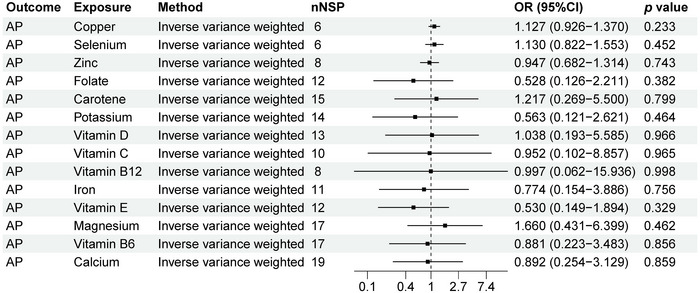
MR analysis results of AP and 14 micronutrients. Forest plot displays the causal associations between acute poliomyelitis (AP) and 14 micronutrients using inverse‐variance weighted (IVW) method. CI: confidence interval; nSNP: number of single‐nucleotide polymorphisms used as instrumental variables; OR: odds ratio.

As shown in Figure 5, we can observe a causal relationship between AP and 14 micronutrients levels. Our study showed that there is no causal relationship between AP and copper, calcium, carotene, folate, iron, magnesium, potassium, selenium, vitamin B12, vitamin B6, vitamin C, vitamin D, vitamin E, and zinc (*p* > 0.05).

### 3.5 MR Analysis Results of SP and 14 Micronutrients

**FIGURE 6:**
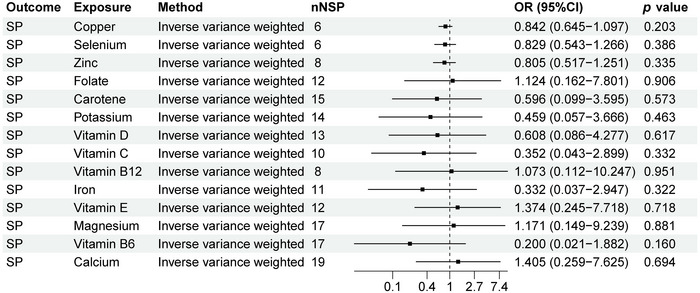
MR analysis results of SP and 14 micronutrients. Forest plot displays the causal associations between sequelae of poliomyelitis (SP) and 14 micronutrients using inverse‐variance weighted (IVW) method. CI: confidence interval; nSNP: number of single‐nucleotide polymorphisms used as instrumental variables; OR: odds ratio.

As shown in Figure 6, we can observe a causal relationship between SP and 14 micronutrients levels. Our study showed that there is no causal relationship between SP and copper, calcium, carotene, folate, iron, magnesium, potassium, selenium, vitamin B12, vitamin B6, vitamin C, vitamin D, vitamin E, and zinc (*p* > 0.05).

### 3.6 MR Analysis Results of ON and 14 Micronutrients

**FIGURE 7:**
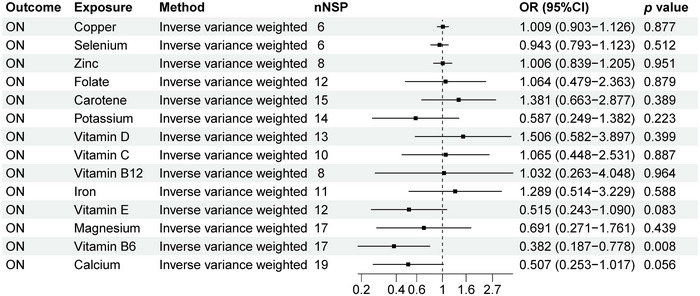
MR analysis results for neuroinflammation. Forest plot displays the causal associations between optic neuritis (ON) and 14 micronutrients using inverse‐variance weighted (IVW) method. CI: confidence interval; nSNP: number of single‐nucleotide polymorphisms used as instrumental variables; OR: odds ratio.

As shown in Figure 7, we can observe a causal relationship between ON and 14 micronutrients levels. There is a causal relationship between ON and vitamin B6 levels (OR: 0.382, 95% CI: 0.187–0.778, *p* = 0.008). The scatter plot in Figure 9C clearly shows the linear relationship between SNP effect values and ON and vitamin B6 levels, suggesting that vitamin B6 levels may be a protective factor for ON. Our study showed that there is no causal relationship between ON and copper, calcium, carotene, folate, iron, potassium, selenium, vitamin B12, vitamin C, vitamin D, vitamin E, and zinc (*p* > 0.05).

### 3.7 MR Analysis Results of MG and 14 Micronutrients

**FIGURE 8:**
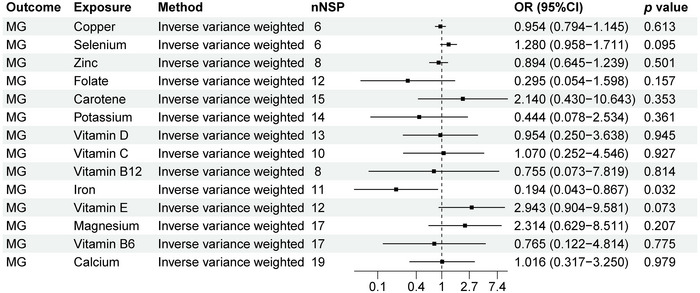
MR analysis results of MG and 14 micronutrients. Forest plot displays the causal associations between myasthenia gravis (MG) and 14 micronutrients using inverse‐variance weighted (IVW) method. CI: confidence interval; nSNP: number of single‐nucleotide polymorphisms used as instrumental variables; OR: odds ratio.

**FIGURE 9:**
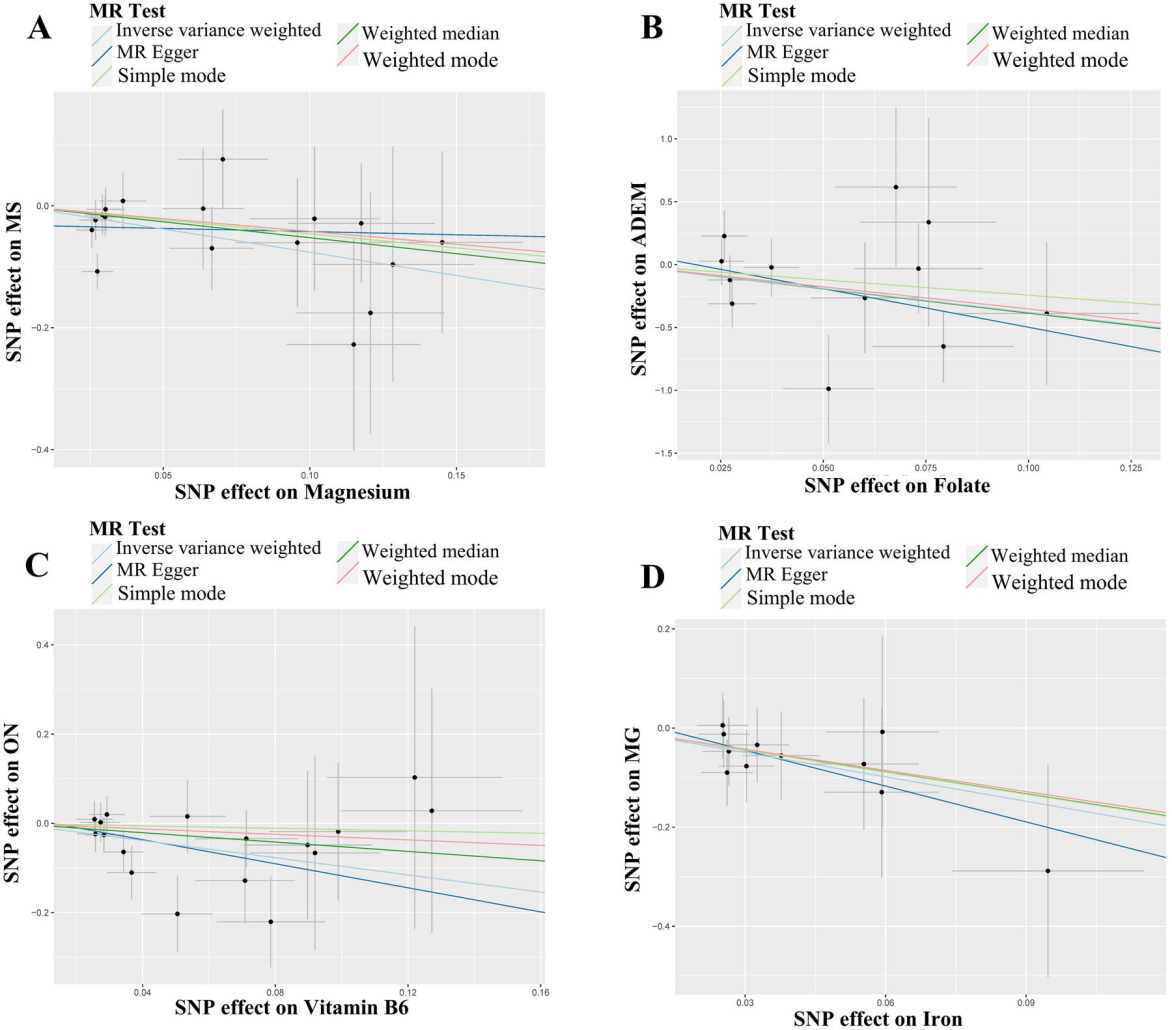
Scatter plot of causality between micronutrient levels and neuroimmune diseases. This figure presents four scatter plots evaluating the causal relationships between micronutrient levels and neuroimmune diseases using Mendelian randomization (MR) analysis. Each point represents a single‐nucleotide polymorphism (SNP) used as an instrumental variable, with regression lines showing the estimated causal effects. *X*‐axis: SNP effect on micronutrient. *Y*‐axis: SNP effect on neuroimmune disease risk. (A) Magnesium versus multiple sclerosis (MS), (B) folate versus acute disseminated encephalomyelitis (ADEM), (C) vitamin B6 versus optic neuritis(ON), and (D) iron versus myasthenia gravis(MG).

As shown in Figure 8, we can observe a causal relationship between MG and 14 micronutrients levels. There is a causal relationship between MG and iron levels (OR: 0.194, 95% CI: 0.043–0.867, *p* = 0.032). The scatter plot in Figure 9D clearly shows the linear relationship between SNP effect values and MG and iron levels, suggesting that iron levels may be a protective factor for MG. Our study showed that there is no causal relationship between MG and copper, calcium, carotene, folate, potassium, selenium, vitamin B12, vitamin B6, vitamin C, vitamin D, vitamin E, and zinc (*p* > 0.05).

### 3.8 Sensitivity Analysis Results of MR

**FIGURE 10:**
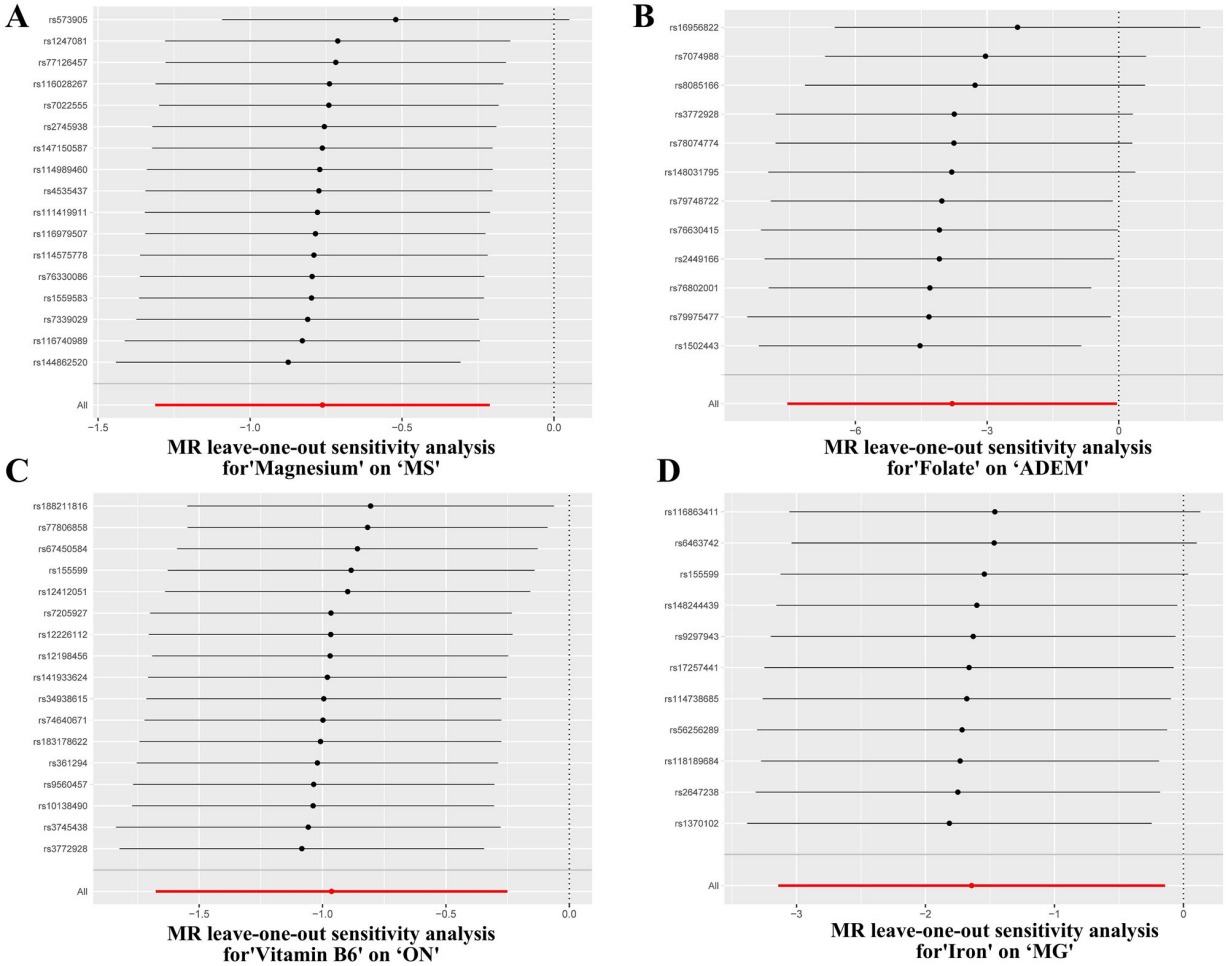
Leave‐one‐out plot to visualize causal effect of micronutrient levels on neuroimmune diseases risk when leaving one SNP out. Leave‐one‐out single‐nucleotide polymorphism sensitivity analysis of micronutrient levels and neuroimmune diseases. The *Y*‐axis represents the IDs of SNPs omitted from the analysis, and the *X*‐axis represents MR odds ratios. The red lines represent MR estimates that include all SNPs. (A) Magnesium versus multiple sclerosis (MS), (B) folate versus acute disseminated encephalomyelitis (ADEM), (C) vitamin B6 versus optic neuritis (ON), and (D) iron versus myasthenia gravis (MG).

We conducted a sensitivity analysis on the results of the MR analysis to ensure the robustness and reliability of the study results (Table 2). We used the IVW test, MR‐Egger's intercept test, and the MR‐PRESSO global test (Table 2). IVW testing based on Cochran's *Q* test showed no heterogeneity (*p* > 0.05), indicating that our analysis results are consistent. In addition, neither the MR‐Egger intercept test nor the MR‐PRESSO global test detected evidence of horizontal pleiotropy (*p* > 0.05), which further strengthens the reliability of our study findings. In addition, the leave‐out‐one analysis shown in Figure 10A–D showed that excluding any single SNP did not significantly change the overall results, confirming the stability and reliability of our results.

**TABLE 2:** Sensitivity analysis results of MR.

Outcome	Exposure	No. of SNP	IVW‐P	OR	95% CI	Horizontal pleiotropy			Heterogeneity	
						Egger intercept	SE	*p* value	Cochran's *Q*	*p* value
MS	Magnesium	16	0.007	0.467	0.269–0.809	−0.0312	0.023	0.184	14.65	0.55
ADEM	Folate	11	0.047	0.022	0.001–0.957	0.116	0.185	0.545	12.164	0.351
ON	Vitamin B6	16	0.008	0.382	0.187–0.778	0.017	0.034	0.616	12.19	0.731
MG	Iron	10	0.032	0.194	0.043–0.867	0.028	0.075	0.723	2.049	0.996

*Note*: This table presents comprehensive sensitivity analyses evaluating the robustness of the Mendelian randomization (MR) findings.

Abbreviations: ADEM: acute disseminated encephalomyelitis; CI: confidence interval; IVW: inverse‐variance weighted; MG: myasthenia gravis; MS: multiple sclerosis; ON: optic neuritis; OR: odds ratio; SE: standard error; SNP: single‐nucleotide polymorphism.

## 4 Discussion

Based on this study, we performed MR analyses of seven neuroimmune diseases and micronutrient levels to assess the causal relationship between disease and micronutrient levels. We found a causal relationship between four neuroimmune diseases (MS, ADEM, ON, MG) and micronutrients levels. The abundance of these micronutrients may have a potential regulatory effect on the occurrence, development, treatment, and prognosis of neuroimmune diseases.

We observed that magnesium is a protective factor in MS. MS is a chronic inflammatory demyelinating disease of the central nervous system, and the immune system is involved in its occurrence and development. MS is a common disabling disease among young people. Its pathogenic characteristics are related to axon degeneration in different areas of the brain and spinal cord and destruction of the blood–brain barrier caused by inflammatory activation (Filippi et al. 2018). Studies have shown that Mg deficiency is associated with neuroimmune regulation (J. A. Maier et al. 2021). Magnesium deficiency promotes the pathogenesis of MS. At physiological concentrations, Mg^2^⁺ exerts a voltage‐dependent blockade of NMDA receptor channels, inhibiting calcium ion influx. Under low magnesium conditions, this blockade is attenuated, leading to glutamatergic signaling hyperactivation, neuronal calcium overload, and excitotoxic damage (Fiorentini et al. 2021) (Mathew and Panonnummal 2021). Concurrently, Mg^2^⁺ acts as an agonist of GABA_A receptors, its deficiency consequently weakens inhibitory neurotransmission, exacerbating neuronal hyperexcitability (J. A. M. Maier et al. 2022). Magnesium deficiency also induces neuroinflammatory responses. A low magnesium environment promotes the polarization of microglia toward the pro‐inflammatory M1 phenotype, characterized by the release of cytokines such as TNF‐α, IL‐6, and increased generation of reactive oxygen species (ROS) (Tsuji et al. 2021). Furthermore, magnesium deficiency upregulates key molecules within the NF‐κB pathway (e.g., TLR4, TRAF6), thereby amplifying the inflammatory cascade and promoting the secretion of additional pro‐inflammatory factors like IL‐8 and MCP‐1 (Almousa et al. 2018a, 2018b). Magnesium deficiency disrupts immune homeostasis. Low magnesium conditions promote the differentiation of Th1/Th17 cells while suppressing the function of regulatory T cells, thereby breaking immune tolerance (Pelczyńska et al. 2022). Mechanisms underlying the protective role of magnesium in MS primarily involve immunomodulation and anti‐inflammatory effects, neuroprotection and antioxidant activity, and blood–brain barrier and myelin protection. In terms of immunomodulatory and anti‐inflammatory effects, studies have found that high concentrations of Mg^2^⁺ block the TLR4/IRAK1/TRAF6 signaling axis, reducing the release of pro‐inflammatory cytokines such as IL‐6 and TNF‐α, thereby mitigating central nervous system inflammation (Ashique et al. 2023). Mg^2^⁺ enhances Treg cell function, inhibits Th17 cell differentiation, and induces B regulatory cells to produce the anti‐inflammatory cytokine IL‐10, promoting immune tolerance (Calahorra et al. 2022). Animal experiments have confirmed that magnesium supplements (such as nano‐MgH_2_) promote the transformation of microglia into anti‐inflammatory M2 type and reduce demyelinating lesions (Z. Li, Chen, et al. 2023). In terms of neuroprotection and antioxidation, Mg^2^⁺ restores the blockade of NMDA receptors, inhibiting calcium influx and protecting neurons from glutamate‐induced excitotoxicity (Lingam and Robertson 2018). Mg^2^⁺ serves as an essential cofactor for the activation of superoxide dismutase, neutralizing ROS and reducing mitochondrial dysfunction and axonal injury (Z. Li, Chen, et al. 2023) (Villa‐Bellosta 2020). In MS animal models, magnesium supplementation restores the expression of synapse‐associated proteins (e.g., PSD95, GluR1), improving synaptic plasticity (Xu et al. 2014). In terms of blood–brain barrier and myelin protection, Mg^2^⁺ enhances blood–brain barrier integrity by upregulating the expression of tight junction proteins, such as Claudin‐5, thereby restricting immune cell infiltration into the central nervous system. Mg^2^⁺ inhibits the TRPM7 channel (involved in calcification signaling), reducing oligodendrocyte apoptosis and promoting myelin regeneration (J. A. M. Maier et al. 2022). Various research signs show that Mg is strongly correlated with the occurrence and development of MS. The current evidence is mostly from animal models and in vitro studies, and more randomized controlled trials in MS patients are needed to verify the efficacy of magnesium supplementation.

Our study found a causal relationship between the trace element folate and ADEM. ADEM is the first demyelinating disease with acute or subacute onset and manifestations of encephalopathy (behavioral abnormalities or disturbance of consciousness) that affects multiple areas of the central nervous system. As a water‐soluble vitamin, folate is necessary for growth and development. Deficiency of folate in the central nervous system can lead to malformations‐neural tube defects during embryonic development and lead to cerebral folate deficiency or folin‐responsive seizures after birth (Djukic 2007). The main incidence of ADEM is children, which may be related to neural tube defects caused by folate. There is currently a lack of relevant literature evidence. However, current studies have shown that when cerebrospinal fluid is extracted to study the effects of folate on the central nervous system, it has been found that folate concentrations may affect oxidative damage, inflammation and NAD (H) levels in the whole body and the central nervous system (Chen et al. 2011) (Czekster et al. 2011). At the metabolic level, as a central cofactor in one‐carbon metabolism, folic acid facilitates the generation of S‐adenosylmethionine (SAM) via its active form, 5‐methyltetrahydrofolate (5‐MTHF). SAM serves as the principal methyl donor, enabling DNA methylation which suppresses the expression of pro‐inflammatory genes (e.g., TNF‐α, IL‐6) in neuroglial cells by promoting promoter methylation (Lan et al. 2012) (C. Li, Ren, et al. 2022). Conversely, folate deficiency results in global DNA hypomethylation, thereby activating inflammatory gene transcription (Kalani et al. 2014). At the redox level, folate deficiency induces homocysteine (Hcy) accumulation. Elevated Hcy activates NMDA receptors, triggering calcium influx and mitochondrial ROS burst. This cascade subsequently activates the NF‐κB pathway and NLRP3 inflammasome, establishing a detrimental “ROS‐inflammation” positive feedback loop (Cheng et al. 2019) (Z. Li, Li, et al. 2022). Folic acid supplementation attenuates Hcy levels, enhances the activity of antioxidant enzymes such as superoxide dismutase and glutathione peroxidase, and mitigates oxidative neuronal damage (Moretti and Caruso 2019). At the immune level, folate deficiency promotes microglial polarization toward the pro‐inflammatory M1 phenotype via the Notch1/NF‐κB p65 pathway, increasing the release of inflammatory mediators (TNF‐α, IL‐1β). In contrast, folic acid supplementation induces the anti‐inflammatory M2 phenotype and inhibits the neurotoxic A1 phenotype in astrocytes (Fan et al. 2015). Furthermore, folic acid enhances Schwann cell proliferation, migration, and secretion of nerve growth factor, facilitating peripheral nerve repair. It also modulates T‐cell differentiation balance (e.g., CD4⁺/CD8⁺ ratio) and augments natural killer cell activity (Kang et al. 2019). And ADEM is a disease characterized by inflammation and myelin destruction. Based on the above literature search and MR analysis, we highly suspect that folate has a certain impact on the neuroimmune disease ADEM.

Our research found that vitamin B6 deficiency has a significant causal relationship with ON. ON is a general term for inflammation of any part of the optic nerve. It generally refers to inflammatory demyelination, infection, non‐specific inflammation, and other diseases of the optic nerve. Vitamin B6 exhibits a complex bimodal relationship with optic neuritis, demonstrating both pathogenic and protective roles contingent upon dosage and metabolic context (Wilmshurst et al. 2019) (Calderon‐Ospina et al. 2020). At supratherapeutic doses, vitamin B6 exerts neurotoxic effects that can precipitate optic nerve injury. The primary mechanism involves its active metabolite pyridoxal 5'‐phosphate (PLP) inducing excitotoxicity through inhibition of pyridoxal kinase (PDXK), which disrupts GABAergic neurotransmission. This preferentially damages large‐diameter sensory nerve fibers, leading to axonal degeneration. Clinical evidence indicates that chronic intake averaging 117 ± 92 mg/day correlates with sensory neuropathy symptoms in 60% of patients, with pathological features consistent with distal axonopathy (Schaumburg et al. 1983). Although optic nerve involvement is less frequent due to its central nervous system designation, experimental studies suggest high‐dose PLP can activate the NF‐κB pathway in astrocytes, amplifying pro‐inflammatory cytokine release and exacerbating optic nerve demyelination and retinal ganglion cell (RGC) apoptosis (Brambilla et al. 2012). Conversely, physiological vitamin B6 levels confer significant neuroprotection through tripartite mechanisms. First, as an essential cofactor for glutathione reductase, it potently scavenges ROS and inhibits lipid peroxidation, with antioxidant efficacy comparable to carotenoids. Primate ischemia‐reperfusion models demonstrate that PLP elevates RGC survival rates to 95% versus 57% in controls, substantially preserving retinal laminar architecture (Wang et al. 2002) (Olivares‐González et al. 2021). Second, it modulates neuroimmune homeostasis by suppressing TLR4/NF‐κB signaling and NLRP3 inflammasome activation, thereby reducing pro‐inflammatory cytokine expression (e.g., TNF‐α, IL‐1β) and mitigating inflammatory infiltration in the optic nerve (Nguyen et al. 2025). Third, it facilitates myelin phospholipid synthesis, maintaining axonal structural integrity. Clinically, supplementation with 450 mg/day vitamin B6 (combined with other B vitamins) for 90 days improved retinal sensitivity thresholds in 76.9% of MS patients, with statistically significant visual function enhancement (*p* = 0.006) (Mallone et al. 2020). The critical determinant of these opposing effects is the serum PLP concentration threshold. Neurotoxicity risk escalates markedly when serum PLP exceeds 68.8 ng/mL, while dietary intake above 1.5 mg/day is associated with reduced risk of optic neuropathies including glaucoma (Yang et al. 2024). Epidemiological studies further indicate that higher dietary vitamin B6 consumption correlates with lower glaucoma incidence among US adults, though serum levels show no consistent association with glaucoma subtypes (Calderon‐Ospina et al. 2020). Genetic factors also modulate this relationship, as PDXK mutations cause autosomal recessive polyneuropathy with optic atrophy that responds to PLP supplementation (Chelban et al. 2019). Therefore, maintaining vitamin B6 within physiological range (RDA: 1.3–1.7 mg/day for adults) is essential to avoid toxicity while preserving neuroprotective benefits (Parra et al. 2018). Particular caution is warranted for high‐risk subgroups, including smokers, the elderly, and oral contraceptive users, who exhibit higher prevalence of suboptimal PLP status even at moderate intakes (Yang et al. 2024). Currently, research on vitamin B6 deficiency primarily focuses on its effects on peripheral nerves or the central nervous system in a broad sense. The optic nerve, as a unique extension of the central nervous system, may exhibit distinct responses to B6 deficiency due to its blood supply and glial cell composition (e.g., oligodendrocytes) (Del Negro et al. 2023). Some studies have found that long‐term B6 deficiency can lead to axonal sensory neuropathy, manifesting as limb numbness and pain, with pathology similar to certain mechanisms of ON (e.g., axonal degeneration) (Muhamad et al. 2023). Additionally, the pathology of optic neuritis involves T‐cell infiltration (particularly CD4+ Th17 cells), leading to demyelination. B6 deficiency may impair regulatory T‐cell function, disrupt immune tolerance, and promote autoimmune attacks (Ciapă et al. 2022). Unfortunately, current research directly exploring the relationship between vitamin B6 deficiency and optic neuritis is limited, and further investigation in this area is needed. Although current research on the direct association between vitamin B6 deficiency and ON remains limited, our genetic evidence has systematically established their causal relationship for the first time. Future studies should focus on (1) establishing ON animal models with B6 deficiency to validate the proposed mechanisms, (2) conducting clinical cohort studies to monitor dynamic changes in serum PLP levels among ON patients, and (3) developing precision B6 supplementation strategies based on genetic risk profiles. These investigations will help elucidate the biological foundations underlying our MR findings and provide novel therapeutic approaches for ON prevention and treatment.

Two sample MR analyses showed that the abundance of trace element iron has a certain impact on MG. MG is an autoimmune disease caused by transmission dysfunction at the neuromuscular junction. The main clinical manifestations are partial or systemic skeletal muscle weakness and fatigue. The symptoms are aggravated after activity and alleviated after rest. Studies have shown that MG is related to serum iron levels, iron metabolism, and iron content (Huang 2023) (k. Li, Hou, et al. 2023) (Colle et al. 1986). Iron exhibits a dual role in the pathogenesis and protection of MG, involving complex mechanisms of immune regulation, oxidative stress, and neuromuscular junction (NMJ) function. In its pathogenic role, iron overload promotes MG development by inducing oxidative stress and ferroptosis. Excess free iron catalyzes fenton reactions, generating ROS that cause lipid peroxidation and cellular damage (Agrawal et al. 2023) (M. H. Choi et al. 2016) (Gomez‐Cabrera et al. 2020). At the NMJ, ROS oxidatively modify myofibrillar proteins (e.g., myosin), impairing their elasticity and contractile function, thereby exacerbating muscle weakness (Beckendorf and Linke 2015). Iron overload also activates microglia to release nitric oxide, dysregulates iron homeostasis proteins, and triggers ferroptosis in motor neurons—a process linked to motor dysfunction in spinal cord injury models and potentially analogous to NMJ transmission defects in MG (Feng et al. 2021). Furthermore, iron overload disrupts immune tolerance by upregulating T‐cell immunoglobulin domain protein 3, suppressing Th1 cell differentiation while promoting the pathogenicity of Th17 cells (Ni et al. 2022) (Pfeifhofer‐Obermair et al. 2021), indirectly facilitating the production of autoantibodies (e.g., anti‐AChR, anti‐MuSK) (Phillips and Vincent 2016). The impact of iron deficiency on MG manifests through multilayered and complex mechanisms. Our MR findings demonstrate that genetically determined iron deficiency exerts protective effects against MG, primarily through immunomodulatory pathways: it reduces pathogenic autoantibody production (e.g., anti‐AChR‐IgG) via B‐cell suppression while disrupting Th17/Treg balance through iron‐sensitive Stat5a/b signaling (Hassan et al. 2016) (Wei et al. 2008). Notably, although iron is essential for mitochondrial function and its deficiency impairs ATP synthesis—a mechanism that would theoretically worsen MG‐related muscle weakness—our genetic evidence suggests this metabolic consequence is clinically outweighed by the dominant immunoprotective effects (Lai et al. 2022) (Alseth et al. 2011). This paradox explains why population‐level data show protection despite observations that some iron‐deficient MG patients exhibit more severe weakness, indicating phenotype‐specific modulation of these competing mechanisms (Thanvi and Lo 2004) (Sekiguchi et al. 2022). The dose‐dependent nature of these effects reveals a therapeutic window: moderate iron restriction may benefit MG through antibody reduction, while severe deficiency risks multisystem dysregulation. This highlights precision iron homeostasis management as a potential intervention strategy. The therapeutic use of iron chelators underscores iron's protective role. In neurodegenerative disorders like Parkinson's disease, deferiprone improves motor function by reducing brain free iron and ROS (Joppe et al. 2019) (Masaldan et al. 2019). Although rare case reports suggest desferrioxamine may directly damage the NMJ causing MG‐like symptoms, most studies indicate that moderate iron chelation mitigates oxidative damage, offering a potential therapeutic strategy for autoimmune diseases like MG (Ma et al. 2021) (Singh et al. 2023).

## 5 Conclusions

Our study demonstrated that micronutrient levels play a crucial role in the onset and progression of neuroimmune diseases. Based on our results and data analysis, specific associations were identified: magnesium with MS, folate with ADEM, vitamin B6 with ON, and iron with MG, all of which significantly contribute to disease pathogenesis. These findings suggest that micronutrients may regulate key factors in neuroimmune disease development. However, the exact mechanisms remain unclear due to a lack of relevant literature, necessitating further experimental research. Thus, our conclusions hold innovative significance.

Understanding the role of micronutrients in diseases may lead to dietary interventions or even pharmaceutical applications for disease management. Clarifying the relationship between micronutrients and neuroimmune diseases is critical—it not only guides micronutrient‐based therapeutic strategies but also provides novel insights into disease mechanisms. This study has several limitations. First, MR examines the causal effect of lifelong genetically determined micronutrient levels on neuroimmune diseases, which differs methodologically from the known physiological roles of these nutrients (e.g., the immunomodulatory effects of vitamins A/D/zinc and the antioxidant functions of vitamins C/E/selenium). Second, the lack of association observed for certain nutrients (e.g., calcium, vitamin B12) may stem from (1) strict homeostatic regulation, resulting in insufficient exposure variation captured by the IVs; (2) the need for multi‐exposure analyses to account for synergistic effects; and (3) statistical power was limited by the current GWAS sample size, particularly for nutrients with smaller effect sizes or weaker instruments. Finally, while the positive findings suggest potential causal mechanisms, experimental studies are required to validate tissue‐specific pathways. Future research should incorporate larger‐scale cohorts and multi‐omics data to further explore these relationships.

## Author Contributions

**Longhao Chen**: conceptualization, methodology, writing – original draft, software. **Xuzhou Wu**: writing – original draft. **Kaizheng Wang**: visualization, investigation. **Xingchen Zhou**: methodology. **Yika Mou**: formal analysis. **Zhen Liu**: visualization. **Zhifang Shen**: writing – review and editing, conceptualization. **Zhizhen Lv**: writing – review and editing, conceptualization. **Lijiang Lv**: writing – review and editing, project administration, supervision.

## Conflicts of Interest

The authors declare no conflicts of interest.

## Peer Review

The peer review history for this article is available at https://publons.com/publon/10.1002/brb3.70848.

## Data Availability

The data that support the findings of this study are available from the corresponding author upon reasonable request.
